# A Case of Acute Pericarditis After COVID-19 Vaccination

**DOI:** 10.3389/falgy.2021.733466

**Published:** 2021-10-01

**Authors:** Andrea Sonaglioni, Adriana Albini, Douglas M. Noonan, Antonio Brucato, Michele Lombardo, Paola Santalucia

**Affiliations:** ^1^Istituto di Ricovero e Cura a Carattere Scientifico (IRCCS) MultiMedica, Milan, Italy; ^2^Scientific and Technological Pole, Istituto di Ricovero e Cura a Carattere Scientifico (IRCCS) MultiMedica, Milan, Italy; ^3^Department of Biotechnology and Life Sciences, University of Insubria, Varese, Italy; ^4^Dipartimento Scienze Biomediche e Cliniche Luigi Sacco, ASST Fatebenefratelli-Sacco, Università degli Studi di Milano, Milan, Italy

**Keywords:** COVID-19, vaccination, Pfizer-BioNTech, acute pericarditis, SARS-CoV-2

## Abstract

A two-dose regimen of Pfizer–BioNTech COVID-19 vaccination confers 95% protection against COronaVIrus Disease 19 (COVID-19) and the safety profile is adequate. To the submission date, there were no reports in literature of acute pericarditis after BNT162b2 vaccination. However, pericarditis has been reported as a rare event associated with COVID-19 infection, which could be due to the pro-inflammatory effects of the spike protein. Recent evidence of post-vaccine myocarditis has been published. Herein we describe the case of a middle-aged healthy women who developed symptoms and signs of acute pericarditis 7–10 days after the second dose of Pfizer–BioNTech COVID-19 vaccination. Although a direct effect cannot be stated, it is important to report a potential adverse vaccine reaction effect that could be associated with the expression of SARS-CoV-2 spike protein induced from the mRNA of the vaccine.

## Introduction

The Pfizer-BioNTech COronaVIrus Disease 19 (COVID-19) mRNA vaccine BNT162b2 is an mRNA-based COVID-19 vaccine, used to provide protection against infection by the Severe Acute Respiratory Syndrome Coronavirus 2 (SARS-CoV-2) Virus in people (1). It is based on the expression of spike RNA which after inoculation expresses the protein and stimulates immune cells to recognize it.

It is known that a two-dose regimen of BNT162b2 confers 95% protection against Covid-19 and the safety profile is adequate (2). The most commonly reported adverse reactions include injection site pain, fatigue, headache, myalgia, chills, arthralgia, Bell's paralysis and fever, and safety aspects are included in the EU's risk management plan (3). Currently, the only relevant side effect is severe allergic reaction (anaphylaxis). To date, there are no reports in literature of acute pericarditis after Pfizer–BioNTech COVID-19 vaccination.

A systematic review reveals a certain number of coronavirus disease 2019 (COVID-19) patients with pericarditis and summarizes the clinical features, diagnostic methods, treatment, and outcomes of the patients (4). Acute pericarditis has often a dubious underlying etiology. In the case of coronavirus disease 2019 (COVID-19) in patients with pericarditis, although there is no definitive test to prove the causal relationship, this complication could be secondary to viral biological effects of COVID-19 infection (4, 5). It is significant to reflect that pericardial disease could be a late complication of COVID-19, and to have therapeutic solutions available when facing such events (4, 5). The major protein involved with SARS-Cov2 pathogenesis is the spike protein, which causes an imbalance of the Renin-Angiotensin-Aldosterone System (RAAS) (6–9).

Herein we present the case of a middle-aged healthy woman who developed symptoms and signs of acute pericarditis 7–10 days after the second dose of Pfizer–BioNTech COVID-19 vaccination. Since the vaccine is based on endogenous spike production, attention should be paid to this possible, although never reported before, complication.

## Clinical Course

A 54-year-old woman, BMI 19.6 Kg/m^2^, without cardiovascular risk factors and with no previous cardiac history, presented to the Emergency Department (ED) with chest pain and simultaneous left upper arm pain, with radiation to the interscapular region, enhanced by inspiration and supine position. These symptoms occurred ~7–10 days after COVID-19 vaccination. The patient had informed consent.

Upon admission, body temperature was 36.5°C, heart rate was 65 beats per minute, blood pressure was 120/80 mmHg, respiratory rate was 15 times per minute, and oxygen saturation was 96% on ambient air. Pericardial friction rubs were not present and respiratory sounds were normal.

Blood tests showed a white blood cell count of 6,650/mmc (63.3% neutrophils and 27.5% lymphocytes), hemoglobin 14.7 g/dl, C-reactive protein (CRP) at the level of 0.03 mg/dl (reference range 0.05–0.50 mg/dl), estimated glomerular filtration rate 81 ml/min/m^2^, and high-sensitive troponin I at the level of 1.45 ng/L (reference range 2.50–38.6 ng/L). Serology for COVID-19 showed an IgG level of 148 AU/ml (protective value >11.5 AU/ml), therefore serology for other viruses was not performed.

**Figure 1:**
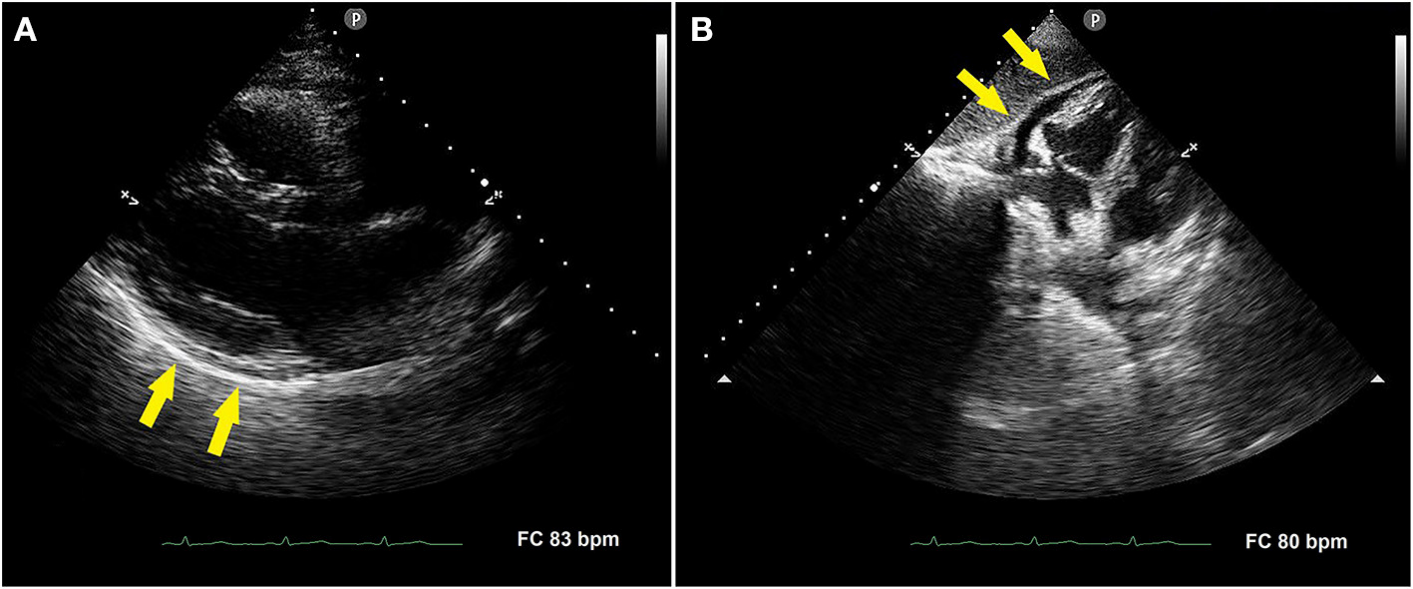
Transthoracic echocardiography performed in the acute phase of disease. Parasternal long-axis view showing mild posterior pericardial detachment [**(A)**, yellow arrows]. Subcostal four-chamber view showing mild anterior pericardial effusion [**(B)**, yellow arrows].

The electrocardiogram (ECG) showed sinus rhythm with normal atrio-ventricular and intra-ventricular conduction, and normal left ventricular repolarization (not shown). Transthoracic echocardiography (TTE) demonstrated mild posterior pericardial detachment (Figure 1A) and mild anterior pericardial effusion (Figure 1B) that was not hemodynamically significant. Cardiac chambers, biventricular systolic function and both left-sided, and right-sided heart valves were normal. Lung ultrasound excluded pleural effusion. Therefore, the patient was diagnosed with acute pericarditis. She received anti-inflammatory treatment with naproxen sodium 550 mg/die and colchicine 1 mg/die, with gradual regression of symptoms.

**Figure 2:**
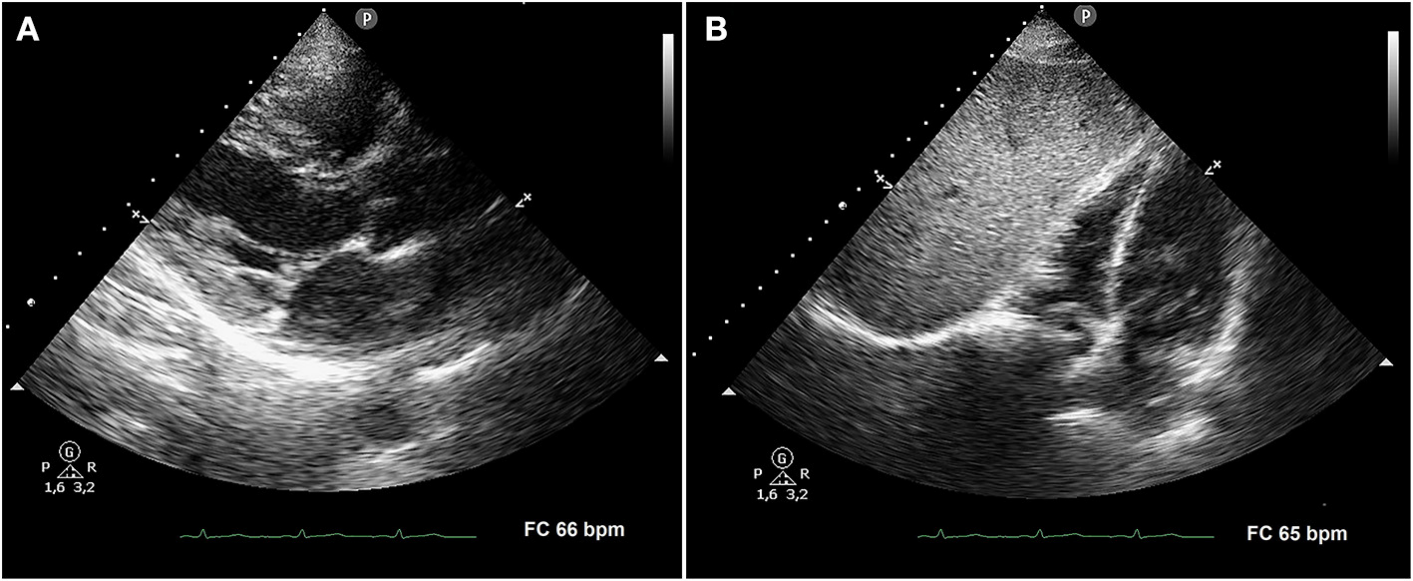
Transthoracic echocardiography performed after 4 weeks of anti-inflammatory treatment. Parasternal long-axis view **(A)** and subcostal four-chamber view **(B)** demonstrating the complete disappearance of both posterior pericardial detachment and anterior pericardial effusion, respectively.

A subsequent TTE, performed after 4 weeks of anti-inflammatory treatment, showed the complete disappearance of both posterior pericardial detachment and anterior pericardial effusion (Figures 2A,B, respectively). Finally, a cardiac magnetic resonance imaging (MRI), performed 40 days after beginning of symptoms, confirmed the complete resolution of pericarditis.

## Discussion

The peculiarity of the present case is that a healthy woman who received a COVID-19 vaccination (with Pfizer-BioNTech COVID-19 mRNA vaccine BNT162b2) developed symptoms and signs of acute pericarditis ~7–10 days after vaccination.

She was diagnosed with acute pericarditis, meeting 2 diagnostic criteria: typical pain and pericardial effusion (10). On the other hand, ECG, inflammatory and myocardial injury indices were normal. The CRP was tested 3 weeks after symptoms onset and this could be the reason for its value within the normal range.

As suggested by the most recent Guidelines (10, 11) anti-inflammatory therapy and colchicine were safe and effective in reducing symptoms. The patient's clinical course was favorable in the absence of any complications such as large pericardial effusion, tamponade, myopericarditis, high inflammatory indices and the response to colchicine treatment was good (12). Cardiac MRI confirmed the complete regression of pericarditis and the absence of myocardial involvement.

To the best of our knowledge, to the submission date, this is the first reported case of acute pericarditis likely related to COVID-19 vaccination in 54 year women. Rare case series of vaccine-related pericarditis have been reported when using mRNA vaccines against COVID-19, particularly in adolescents and young adults (<30 years) and mainly male; symptom onset is usually within 1 week following vaccination (13–15). Clinical course appears to be mild in most cases, these patients can usually return to their normal daily activities after their symptoms improve (13, 14). A study in forty hospitals in Washington, Oregon, Montana, and Los Angeles County (individuals 2000287 least 1 COVID-19 vaccination) showed 37 vaccinated patients who subsequently had diagnoses of acute pericarditis, mostly were male (73%), and the median age was 59 years, 13 patients were admitted to the hospital, none to intensive care and none of them died (16).

The correlation of typical chest pain and pericardial effusion with COVID-19 vaccination was “suspected” given the absence of other etiologic findings, the temporal relationship to the vaccination, and positive IgG anti-COVID-19 serology. Myocarditis and pericarditis represent serious and life-threatening inflammatory diseases involving myocardium and pericardium, potentially associated with the use of several drugs and vaccines (10, 17, 18). Rare cases of myo-pericarditis after influenza immunization have been reported (19, 20).

In the present case, COVID-19 mRNA vaccine may have triggered an inflammatory response that was responsible for pericardial injury. In particular, the mRNA vaccine might have induced a molecular mimicry mechanism between the viral spike protein and an unknown cardiac protein (21, 22). The vaccine is based on SARS-CoV-2 spike RNA to synthesize the protein antigen. Spike, besides to ACE2 (6–9), binds the toll-like receptor TLR4 (23), and spike protein binds to bacterial lipopolysaccharide (24, 25). The high anti-spike IgG antibody titer and the good response to anti-inflammatory therapy and colchicine suggested the possible immune-mediated etiology of pericarditis (26).

The patient's age (54 years old) was in alignment with that observed in the great majority of COVID-19 related pericarditis (4). Moreover, the present case revealed that the occurrence of a COVID-19 vaccine related cardiac involvement in the absence of pulmonary findings is possible.

In the present case, the normal serum level of high-sensitive troponin I, the normal cardiac chambers size and the normal biventricular systolic function assessed by TTE, allowed to exclude any myocardial injury. On the other hand, the pericardial effusion with normality of the remaining echocardiographic findings suggested exclusive pericardial involvement with no evidence of myocarditis. The diagnosis of pericarditis was formulated on the basis of two diagnostic criteria: (1) the typical chest pain and (2) the pericardial effusion.

To date, cardiac injury (troponin I elevation, ECG and echocardiography abnormalities) has been observed in ~7.2% of patients with severe and critical COVID-19 infection (27–33), while pericardial effusion was found in 4.8% of patients, which suggests that acute pericarditis could be an underdiagnosed pathology, and therefore, not properly managed and treated (5, 34, 35).

The pathogenesis of cardiac involvement associated with SARS-CoV-2 may reflect a process of replication and dissemination of the virus through the blood or the lymphatic system from the respiratory tract. Alternatively, SARS-CoV-2 could trigger an exaggerated inflammatory response that can cause myocardial injury (6, 36). Finally, a potential binding to a viral receptor of the myocyte can favor the internalization and subsequent replication of the capsid proteins and the viral genome (37, 38).

Currently, a validated test to assess COVID-19 in the pericardial fluid need to be developed. COVID-19 studies are needed to determine in pericardial fluid and how to correctly check for its occurrence (35).

## Conclusion

A cardiac involvement in the absence of pulmonary findings could be suspected in symptomatic subjects who have undergone COVID-19 vaccination. Acute pericarditis is often underdiagnosed. The occurrence of an acute pericarditis related to a COVID-19 vaccination may be underpinned by an immune-mediated mechanism.

## Data Availability Statement

The raw data supporting the conclusions of this article will be made available by the authors, without undue reservation.

## Ethics Statement

Ethical review and approval was not required for the study on human participants in accordance with the local legislation and institutional requirements. The patients/participants provided their written informed consent to participate in this study. Written informed consent was obtained from the individual for the publication of any potentially identifiable images or data included in this article.

## Author Contributions

AS, ML, and PS: conceptualization. AS, ML, and AB: performed the clinical testing of the patient. AS, ML, AB, PS, DN, and AA: analyzed data. AS, AA, DN, ML, and PS: data curation. AS, ML, and PS: writing original draft preparation. AS, AA, and DN: writing review and editing. AA and DN: funding acquisition. All authors have read and agreed to the published version of the manuscript.

## Funding

This research was funded by a grant of the Ministero della Salute COVID-2020-12371849, to DN. This work has also been supported by the Italian Ministry of Health Ricerca Corrente—IRCCS MultiMedica.

## Conflict of Interest

The authors declare that the research was conducted in the absence of any commercial or financial relationships that could be construed as a potential conflict of interest.

## Publisher's Note

All claims expressed in this article are solely those of the authors and do not necessarily represent those of their affiliated organizations, or those of the publisher, the editors and the reviewers. Any product that may be evaluated in this article, or claim that may be made by its manufacturer, is not guaranteed or endorsed by the publisher.
